# Optimization of ultrasonic-assisted debittering of *Ganoderma lucidum* using response surface methodology, characterization, and evaluation of antioxidant activity

**DOI:** 10.7717/peerj.17943

**Published:** 2024-10-14

**Authors:** Shuting Chen, Shiying Song, Yumei Tan, Shengling He, Xiyi Ren, Zhu Li, Yongxiang Liu

**Affiliations:** 1 Key Laboratory of Plant Resource Conservation and Germplasm Innovation in Mountainous Region (Ministry of Education), College of Life Sciences/Institute of Agro-bioengineering, Guizhou University, Guiyang, Guizhou Province, China; 2 Guizhou Academy of Agricultural Sciences, Guizhou Key Laboratory of Agricultural Biotechnology, Guiyang, Guizhou Province, China; 3 Ministry of Agriculture and Rural Affairs Key Laboratory of Crop Genetic Resources and Germplasm Innovation in Karst Region, Guiyang, Guizhou Province, China

**Keywords:** Ganoderma lucidum, Debittering, Characterization, Antioxidant activity, Response surface methodology, Spray drying, Freeze drying

## Abstract

**Background:**

*Ganoderma lucidum* (*G. lucidum*) has gained increasing attention as a potential health care product and food source. However, the bitter taste of *G. lucidum* has limited its development and utilization for the food industry.

**Methonds:**

The response surface methodology was employed to optimize the inclusion conditions for the debittering of *G. lucidum*. The effects of 2-hydroxypropyl-*β*-cyclodextrin concentration (12–14 g/mL), ultrasound temperature (20–40 °C and host—guest ratio (1:1–2:1) on response variables were studied. The physical characteristics of inclusion complexes prepared through spray drying and freeze drying were analyzed. The antioxidant activity of the different treated samples was subsequently investigated.

**Results:**

Study results showed that, in comparison to the control group, the inclusion solution displayed a significantly enhanced taste profile under optimal processing conditions, exhibiting an 80.74% reduction in bitterness value. Fourier transform infrared spectroscopy (FTIR) and proton nuclear magnetic resonance (NMR) studies indicated the successful formation of inclusion compounds. The moisture content and bulk density of spray-dried powder were found to be significantly superior to those of freeze-dried powder (*p* < 0.05). In comparison to the diluted solution, the inclusion liquid demonstrated a 20.27%, 30.01% and 36.55% increase in ferric ion reducing antioxidant power (FRAP), hydroxyl radical scavenging and 2,2-diphenyl-1-picrylhydrazyl radical (DPPH) scavenging respectively. Further, the DPPH clearance of microencapsulated powder was not significantly different from that of tocopherol at a concentration of 25 mg/mL.

**Conclusions:**

In summary, the study provides theoretical basis and methodological guidance to eliminate the bitterness of *G. lucidum*, and therefore provide potential options to the use of *G. lucidum* as a food source.

## Introduction

*Ganoderma lucidum* is a medicinal mushroom that has been used for many millennia in the Far East due to its efficacious medicinal properties (Cancemi et al., 2024; Cör Andrejč, Knez & Knez Marevci, 2022; Feng et al., 2017). It contain various biologically active compounds, including polysaccharides, triterpenoids, steroids, amino acids, nucleotides, and alkaloids (Cör, Knez & Hrnči, 2018; Oke et al., 2022). These compounds have been demonstrated to strengthen the immune system and confer a range of health benefits, including antitumor and antioxidant effects, as well as liver protection (Cör Andrejč, Knez & Knez Marevci, 2022; Ekiz et al., 2023; Kolniak-Ostek et al., 2022). The prevalence of dietary therapies is on the rise, with an increasing number of individuals turning to these approaches to maintain their health and to prevent or treat cancer. This trend aligns with the growing trend towards natural living (Tan et al., 2022). The inclusion of *G. lucidum* in China’s 2023 list of homologues for medicinal and food products has the potential to significantly impact the market development. Nevertheless, the bitter taste of *G. lucidum* has been a limiting factor in its acceptance as a food product. Consequently, *G. lucidum* is frequently transformed into diverse products, including powders, dietary supplements, and herbal tea (Bishop et al., 2015; Bryant, Bouchard & Haque, 2017; El Sheikha & Hu, 2018; El Sheikha, 2022), with the objective of improving the palatability of the final products. The principal bitter compounds in *G. lucidum* are triterpenoids, which have been demonstrated to possess multiple beneficial properties, including anticancer and antimicrobial effects (Ahmad et al., 2023; Galappaththi et al., 2022). To retain the potential health benefits of unstudied bitter substances, it is advisable to form an inclusion rather than removing them directly (Deshaware et al., 2018).

Cyclodextrins (CDs) are cyclic oligosaccharides derived from starch. They possess a lipophilic central cavity and a hydrophilic outer surface (Hadian et al., 2023; Prodea et al., 2022). The complexation of CDs improves the stability of encapsulated components and food products against light, heat, pH, ionisation state, oxidation, volatility, browning and microbial contamination (Jo, Cho & Chun, 2021). *β*-Cyclodextrin (*β*-CD) is a widely used compound in the food and pharmaceutical industries due to its ability to form stable inclusion complexes (ICs) with hydrophobic molecules (Jiang et al., 2021). Deshaware et al. (2018) demonstrated that an IC is formed through the interaction of the hydrophobic triterpenoidal region of momordicosides with *β*-CD, thereby enhancing the palatability of bitter gourd juice. The poor aqueous solubility of native *β*-CD presents a significant challenge to its utilization. Hydroxypropyl-*β*-cyclodextrin (HP-*β*-CD) is a modified version of *β*-CD that exhibits markedly enhanced water solubility and complexation efficiency in comparison to the native *β*-CD (Cui, Siva & Lin, 2019).

The use of activity compounds is constrained by their instability in conditions of high temperature, oxygen presence, light exposure, enzymatic activity, and pH variations (Valková et al., 2022). To enhance their stability and overcome these limitations, microencapsulation techniques such as freeze drying (FD) and spray drying (SD) can be employed (Buljeta et al., 2022; Zhao et al., 2021). CDs have been considered as one of the simplest and most effective wall materials for encapsulation (Jiang et al., 2021). Due to its low-temperature treatments, FD is a more appropriate method for encapsulating heat-sensitive compounds and materials. However, the process is disadvantageous in terms of lengthy drying times, high energy consumption, and high unit costs (Yaman et al., 2023; Zhao et al., 2021). Conversely, SD offers a number of advantages, including straightforward adjustment and control, minimal processing costs, and uninterrupted operation. Nevertheless, the processing temperature is typically above 100 °C, which can result in the degradation of heat-sensitive substances (Buljeta et al., 2022; Zhao et al., 2021). The stability of microencapsulated powders (MPs) is significantly influenced by a number of factors, including moisture content, bulk density, solubility, and hygroscopicity (Ermis & Özkan, 2020). This information can be utilized to select appropriate storage conditions and packaging systems that enhance the preservation of flavor, nutrients, bioactive components, and physical stability (Franceschinis et al., 2013; Liu et al., 2024).

The removal of bitterness from *G. lucidum* decoction has, hitherto, rarely been reported in current literatures. Consequently, the study employed response surface methodology (RSM) for the purpose of optimizing the process of debittering bitterness and investigating the interactive effects among the independent variables. RSM is a statistical technique that can determine the ideal experimental conditions with minimal trials (Baca-Bocanegra et al., 2021; Bukhari et al., 2022). The synthesis of the inclusion complex of a bitter substance/2-HP- *β*-CD was successfully achieved through the application of ultrasonic waves. A further challenge is to enhance biological activity while improving palatability. Furthermore, the preparation of microencapsulated powder by SD and FD technology can extend the shelf life, thereby providing greater potential for *G. lucidum* in food applications.

## Materials and Methods

### Raw materials and reagents

The *G. lucidum* samples were procured from Xiancao Biotechnology Co. (Shandong, China), while the 2-hydroxypropyl- *β*-cyclodextrin (2-HP-*β*-CD, food grade) was obtained from Huaxing Biological Chemical Co. (Henan, China). The ferric ion reducing antioxidant power (FRAP), hydroxyl free radical, and 2,2-diphenyl-1-picrylhydrazyl radical (DPPH) test kits were obtained from Gris (Suzhou, China). All other chemicals employed in this study were of analytical grade.

### Preparation of samples

The appropriate amount of 2-HP-*β*-CD was measured and added to distilled water. The mixture was homogenized using sonication until a clear aqueous 2-HP-*β*-CD solution (host) was formed. Next, the *G. lucidum* pieces were immersed in distilled water for 30 min, followed by two half-hour decoctions. The *G. lucidum* decoction (guest) was obtained by filtering and combining the resulting filtrates. The inclusion liquid was created by mixing the host and guest solutions and applying ultrasound treatment. A dilution was achieved by combining the decoction of *G. lucidum* with a specific quantity of distilled water.

### Experimental design

The debittering conditions have been optimized by means of a Box-Behnken design (BBD) (Baca-Bocanegra et al., 2021; Bukhari et al., 2022). The independent variables and their levels were as follows: the concentration of 2-HP-*β*-CD, which ranged from 10 to 14 g/mL; the ultrasonic temperature, which ranged from 20 to 40 °C; and the host-guest ratio, which varied from 1:1 to 2:1. The dependent variable (bitterness value) was considered as the response. Table 1 presents the coded levels of each factor, representing both the range and the midpoint values for these three independent variables. A total of seventeen experiments were conducted in triplicate, in accordance with the procedure that outlined in Table 2. The generalized RSM model for expressing the variation in the response variable as a function of the independent variable is summarized in the following equation:

(1) \begin{eqnarray*}Y={a}_{0}+{a}_{1}A+{a}_{2}B+{a}_{3}C+{a}_{11}{A}^{2}+{a}_{22}{B}^{2}+{a}_{33}{C}^{2}+{a}_{1}{a}_{2}AB+{a}_{1}{a}_{3}AC+{a}_{2}{a}_{3}BC+E.\end{eqnarray*}

In the provided equation, *Y* represents the percentage of yield response. The variables A, B, and C indicate the 2-HP-*β*-CD concentration, temperature and host-guest ratio respectively. Meanwhile, a_0_ is referred to as the intercept while a_1_, a_2_, and a_3_ are coefficients representing linearity. Furthermore, a_11_, a_22_,and a_33_ represent quadratic coefficients whereas interactions are denoted by a_1_a_2_, a_1_a_3_, and a_2_a_3_. The symbol E represents the error function in this model.

**Table 1:** Response surface design independent variables and their levels.

Independent variables	Coded symbols	Coded factor levels		
		−1	0	1
2-HP- *β*-CD concentration (g/L)	A	10	12	14
Temperature (°C)	B	20	30	40
Host-Guest ratio (x:1)	C	1	1.5	2

**Table 2:** Experimental design for removing bitterness with independent variables, experimental and predicted responses.

Run	A (g/L)	B (°C)	C (x:1)	Bitterness value	
				Experimental	Predicted
1	12	30	1.5	1.64 ± 0.10	1.68
2	14	40	1.5	1.59 ± 0.17	1.61
3	12	30	1.5	1.69 ± 0.10	1.68
4	12	40	1	1.97 ± 0.17	1.96
5	10	20	1.5	2.23 ± 0.47	2.21
6	10	40	1.5	2.22 ± 0.45	2.24
7	10	30	2	2.01 ± 0.16	2.02
8	12	30	1.5	1.73 ± 0.14	1.68
9	14	30	2	1.58 ± 0.10	1.59
10	12	40	2	1.56 ± 0.13	1.53
11	12	30	1.5	1.75 ± 0.23	1.68
12	14	20	1.5	1.62 ± 0.20	1.60
13	14	30	1	1.81 ± 0.19	1.80
14	12	20	1	1.89 ± 0.31	1.92
15	10	30	1	2.63 ± 0.35	2.62
16	12	30	1.5	1.60 ± 0.16	1.68
17	12	20	2	1.51 ± 0.10	1.52

### Model validation

Subsequent experiments were conducted utilising the optimal parameters in order to validate the model derived from RSM.

### Electronic tongue test

The test procedure utilized an electronic tongue (SA402B, Insent, Kanagawa, Japan) and involved a series of sequential actions, including cleaning, calibration and data acquisition of both sample and reference solutions (Zhang et al., 2018). The sensor was submerged in the solution for a period of 90 s in order to facilitate the removal of impurities from the surface of the film. Following a further 30 s of stabilization in the reference solution, the potential value (Vr) was then measured. Subsequently, the sensor was maintained in the sample solution for a further 30 s, resulting in the recorded potential value (Vs). The potential difference V_1_ was calculated as Vs-Vr and represents the output value of the base flavour.

### Preparation of microencapsulated powder

The MP were prepared in accordance with the methodology outlined by Pereira et al. (2022), with certain modifications. The inclusion liquid was stored at −20 °C for 12 h and subsequently subjected to lyophilisation for 24 h. The freeze-dried powder (FDP) was then dissolved in dimethyl sulfoxide (DMSO) for subsequent analysis.

The inclusion liquid and *G. lucidum* decoction were subjected to drying using a laboratory-scale apparatus (B-290, Büchi, Switzerland). Throughout the course of this process, the aspirator and pump capacities were maintained at their maximum levels, specifically 100% and 18%, respectively. A temperature of 180 °C was selected in order to achieve an outlet temperature of approximately 108 °C (Čulina et al., 2023). The resulting spray-dried powder (SDP) and *G. lucidum* powder (GLP) was subsequently stored in glass bottles for further analysis in accordance with the experimental protocol.

### Characterization analysis

#### Fourier-transform infrared

The methodologies described in the references were modified to a limited extent (Cui, Siva & Lin, 2019; Hadian et al., 2023). Infrared spectroscopy (Nicolet 6700, Thermo Fisher Scientific, Waltham, MA, USA) was employed to obtain the infrared spectra of the samples at room temperature. The KBr was combined with 2-HP-*β*-CD, GLP, FDP and SDP in a 1:100 ratio for the purpose of tabletting. The wave number range was set at 400 to 4,000 cm^−1^, with a scanning time of 32 s.

#### Nuclear magnetic resonance

A few minor alterations were made to the methodology outlined in the referenced article (Deshaware et al., 2018; Hadian et al., 2023). The nuclear magnetic resonance (NMR) experiments were conducted using a spectrometer (600 MHz, Bruker, Germany) with a proton frequency of 600.13 MHz and a five mm TXI probe at a temperature of 25 °C. Proton NMR spectra were obtained with a spectral width of 14,705.9 Hz, an acquisition time of 2.7525 s, and a relaxation delay of 1 s. Each sample was scanned eight times in total.

### Microencapsulated powder characterization

#### Moisture content

Moisture content was determined for both GLP, FDP and SDP samples using a moisture analyzer (Youke, Shanghai, China) at a temperature of 102 ±2 °C (Čulina et al., 2023).

#### Hygroscopicity

The study evaluated the moisture absorption of GLP, FDP and SDP using a modified version of the methodology described by (Čulina et al., 2023; Tonon, Brabet & Hubinger, 2008). Specifically, 1 g of powder was evenly distributed on a pre-weighed glass Petri dish and placed in a desiccator containing 100 mL of a saturated sodium chloride solution (74.3% humidity). Following a seven-day period, the powders were reweighed in order to ascertain their hygroscopicity, expressed as the quantity of water adsorbed per 100 g of powder (g/100 g).

(2) \begin{eqnarray*}\text{Hygroscopicity}(g/100g)= \frac{{m}_{7}-{m}_{0}}{{m}_{0}} \times 100.\end{eqnarray*}

The variable m_7_ represents the mass (in grams) of the powder after a storage period of seven days, while m_0_ denotes the initial mass (in grams) of the powder prior to storage.

#### Solubility

The solubility of the MPs was determined using the method described in the reference (Čulina et al., 2023; Gawałek, 2022). Approximately 0.1 g of powder was added to 10 mL of water, and the resulting mixture was agitated (78-1, Jerrell, Jiangsu, China) at 700 r/min for 5 min and subsequently centrifuged at 4,000 r/min for 10 min. The resulting supernatant was collected, dried to a constant mass in a laboratory oven maintained at 55 °C, and used to calculate the percentage solubility using the provided equation.

(3) \begin{eqnarray*}\text{Solubility}(\%)= \frac{{m}_{1}}{{m}_{0}} \times 100.\end{eqnarray*}

The variable m_1_ represents the mass (in grams) obtained through desiccation of the liquid residue, while m_0_ refers to the mass (in grams) of the substance utilised in the analysis.

#### Bulk density

The bulk densities of the MPs were determined using the method described in the referenced source (Čulina et al., 2023; Rodríguez-Cortina, Rodríguez-Cortina & Hernández-Carrión, 2022). In order to ensure uniform particle dispersion, the powders were transferred into a test tube and subjected to a 60 s vibration cycle on a vortex vibrator. The volume of powder was determined by positioning the tube on a stable surface, and the ratio of powder mass to its corresponding volume in millilitres (g/mL) was calculated to obtain the respective bulk densities.

### Antioxidant activity assay

The antioxidant properties of GLP, FDP and SDP were evaluated through *in vitro* assays, including DPPH and hydroxyl radical scavenging activities, as well as a FRAP assay to measure reduction power. All assays were conducted using the microplate reader (Epoch2, BioTek Instruments, Inc).

#### Ferric reducing antioxidant power assay

The capacity of the sample to reduce the ferric tripyridyltriazine complex was evaluated using a slightly modified experimental method (Marčetić et al., 2022; Wang et al., 2023). Absorbance measurements were conducted at a wavelength of 590 nm. A calibration curve was constructed utilizing a standard FeSO_4_ solution spanning a range of 1 to 5 µmol/mL, with *α*-Trolox employed as the positive control. The results are reported in µmol FeSO_4_/mL.

#### Hydroxyl radical scavenging assay

Hydroxyl radicals are observed in the fenton reaction when it occurs in conjunction with reduced transition metal ions, such as Fe^2+^, and hydrogen peroxide (H_2_O_2_). A slightly modified version of the previously reported method (Xie et al., 2020) was employed to obtain data at 510 nm, with *α*-Trolox serving as the positive control. The results are presented as clearance expressed as a percentage.

#### DPPH radical scavenging capacity assay

The DPPH radical scavenging capabilities were evaluated using a previously outlined method, with certain modifications incorporated (Jeon et al., 2022). Absorbance measurements were taken at 517 nm with the objective of establishing a calibration curve, using Trolox (5–25 µg/mL) as the standard. The positive control was *α*-trolox. A reduction in the absorbance within the reaction mixture indicated an enhanced capacity for the scavenging of free radicals. The results are presented as a percentage clearance.

### Statistical analysis

The mean and standard deviation were presented for the data set, which consisted of three observations. A statistical analysis was conducted using analysis of variance (ANOVA) with a significance level of *p* < 0.05. The statistical software employed for the analysis included SPSS 26.0 and Design Expert 10, while Origin 2018 was utilized to generate the graphs.

## Results and Discussion

### Process optimization for debittering

#### Model fitting

The statistical model demonstrated high accuracy with no significant lack of fit (*p* ≤ 0.05) for all variables listed in Table 3. A higher coeffcient of determination (R^2^) value indicates better alignment between the model and experimental data, while a lower R^2^ value suggests insufficient explanation of behavioural variations (Li & Chiang, 2012; Mehmood et al., 2018). Our study demonstrated that the quadratic polynomial model accurately describes 2-HP-*β*-CD concentration (A), temperature (B), and host-guest ratio (C). The significance level of coefficients within this model was determined using ANOVA testing. Larger *F*-values and smaller *p*-values indicate highly significant effects on response variables (Mehmood, 2015; Mehmood et al., 2019).

**Table 3:** Analysis of the variance of the fitted second-order polynomial models.

Source	Sum of squares	Df	MS	*F*-value	*P*-value	Significance
Model	1.45	9	0.16	57.78	<0.0001	^**^
A	0.78	1	0.78	277.43	<0.0001	^**^
B	1.013E−003	1	1.013E−003	0.36	0.5661	
C	0.34	1	0.34	120.35	<0.0001	^**^
AB	1.000E−004	1	1.000E−004	0.036	0.8553	
AC	0.038	1	0.038	13.61	0.0078	^**^
BC	2.250E−004	1	2.250E−004	0.081	0.7848	
A^2^	0.27	1	0.27	97.24	<0.0001	^**^
B^2^	1.857E−003	1	1.857E−003	0.66	0.4418	
C^2^	0.022	1	0.022	7.71	0.0275	^*^
Residual	0.020	7	2.794E−003			
Lack of Fit	4.075E−003	3	1.358E−003	0.35	0.7919	Not significant
Pure Error	0.015	4	3.870E−003			
Cor Total	1.47	16				
*R*^2^ = 0.9867	R^2^_Adj_ = 0.9696	R^2^_Pred_ = 0.9393				
C.V% = 2.90	Adeq Precision = 27.074					

**Notes.**

* *p* < 0.05, the difference is significant at 0.05 level.

** *p* < 0.01, the difference is extremely significant at 0.01.

The R^2^ and the adjusted coefficient of determination (R^2^_adj_) were both approximately equal to 1, indicating a high accuracy in predicting bitterness values, and a strong agreement between experimental and predicted values. Additionally, it is important to note that the coefficient of variation (CV) remained below 10%, indicating excellent precision and reproducibility within the model’s performance. The results indicate that the mathematical model accurately calculated bitterness values. Based on the F value, the bitterness value is influenced by the test conditions in the following order: A > C> B. The bitter taste of the liquid is primarily determined by variables A and C, which have significant linear (*p* < 0.0001), quadratic (*p* < 0.0001), and interactive (*p* < 0.05) effects. These results are summarized in Table 3. The quadratic model expressions below can be used to predict the bitterness value response.

(4) \begin{eqnarray*}{Y}_{\mathrm{(bitternessvalue)}}=1.67-0.31A+0.016B-0.20C-2.500E-003AB+0.098AC\nonumber\\\displaystyle -3.750E-003BC+0.25{A}^{2}-5.25E-003{B}^{2}+0.071{C}^{2}.\end{eqnarray*}

#### Response surface analysis

**Figure 1:**
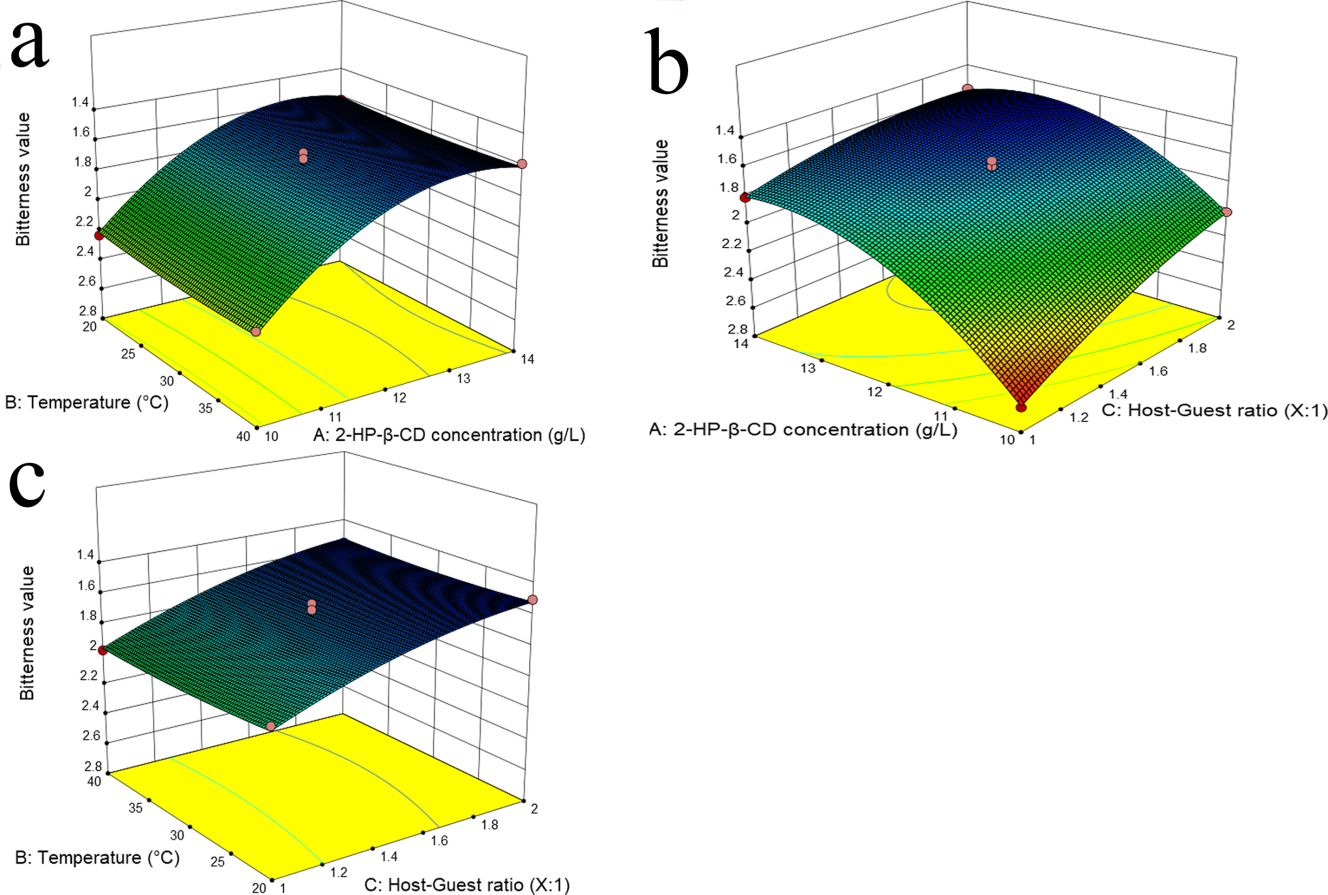
Response surface plots of independent variables for the bitterness showing the minimum for the variables combination.

The study employed surface response plots, created using a quadratic polynomial model, to analyze the effect of independent variables on the dependent variable (Bukhari et al., 2022; Li & Chiang, 2012). Figures 1A, 1B demonstrates that the bitterness value initially decreased and then increased with the progressive increase in 2-HP-*β*-CD concentration. One potential explanation for this phenomenon is that as the concentration of 2-HP-*β*-CD increases, there is a greater probability of collisions between bitter molecules and 2-HP-*β*-CD molecules, which may result in a reduction in perceived bitterness. Nevertheless, a high concentration of the 2-HP-*β*-CD solution may result in intermolecular adhesion due to its physicochemical properties (Yinghui, 2021). This can result in a reduction in the proportion of 2-HP-*β*-CD molecules that encapsulate bitter molecules, which subsequently increases bitterness. The application of ultrasonication has been demonstrated to effectively reduce the intermolecular adhesion between 2-HP-*β*-CD molecules, thereby promoting homogeneity and enhancing the formation rate of ICs 48 (Mongenot, Charrier & Chalier, 2000). As illustrated in Figs. 1B, 1C, the perception of bitterness is more pronounced when the ratio of 2-HP-*β*-CD is less than that of the bitter substance. The relatively large number of bitter molecules causing collisions is responsible for the reduced entry of these molecules into the 2-HP-*β*-CD cavity. Additionally, due to a high presence of molecules in the solution system, smaller molecules may adsorb onto larger 2-HP-*β*-CD molecules through hydrogen bonding and intermolecular forces (Baoqiang, 2021). As shown in Table 3 and Figs. 1A, 1C temperature did not significantly influence the reaction, suggesting that it can be effectively carried out within a range of 20−40 °C. To ensure successful formation of ICs, it is important to avoid excessively high reaction temperatures. This is because the process involves an exothermic reaction (Ning, 2015), and as the temperature increases, molecular motion accelerates, reducing the collision between guest and host molecules and hindering encapsulation.

### Experimental validation of optimized conditions

As illustrated in Table 4, in order to facilitate practical application, the experimental conditions were subject to a slight adjustment. The percentage difference between the predicted optimal values and the experimental ones was only 6.5%, which is considered low in the desired region where the responses were maximized (Favre et al., 2018). These results prove the effectiveness of using experimental planning such as RSM and the desirability function approach to optimize multiple response variables. This model enables the investigation of optimal experimental conditions to attain the desired level of bitterness.

**Table 4:** Verification results (*n* = 3).

Group	A (g/L)	B (x:1)	C (° C)	Bitterness value
Prediction	12.876	1.878	35.161	1.509^b^
Experimental	13	1.9	35	1.411 ± 0.208^b^
Original	–	–	–	7.316 ± 0.231^a^

**Notes.**

Means with different lowercase display significant differences (*p* < 0.05).

### Electronic tongue flavor analysis

Electronic tongues, which mimic human taste receptors, are used for sensory evaluation and reduce subjectivity in taste assessment (Jiang et al., 2018). Researchers employ sensor technology to analyze liquid samples qualitatively or quantitatively by measuring the overall electrical signal strength associated with them, thereby reflecting their comprehensive taste characteristics.

**Figure 2:**
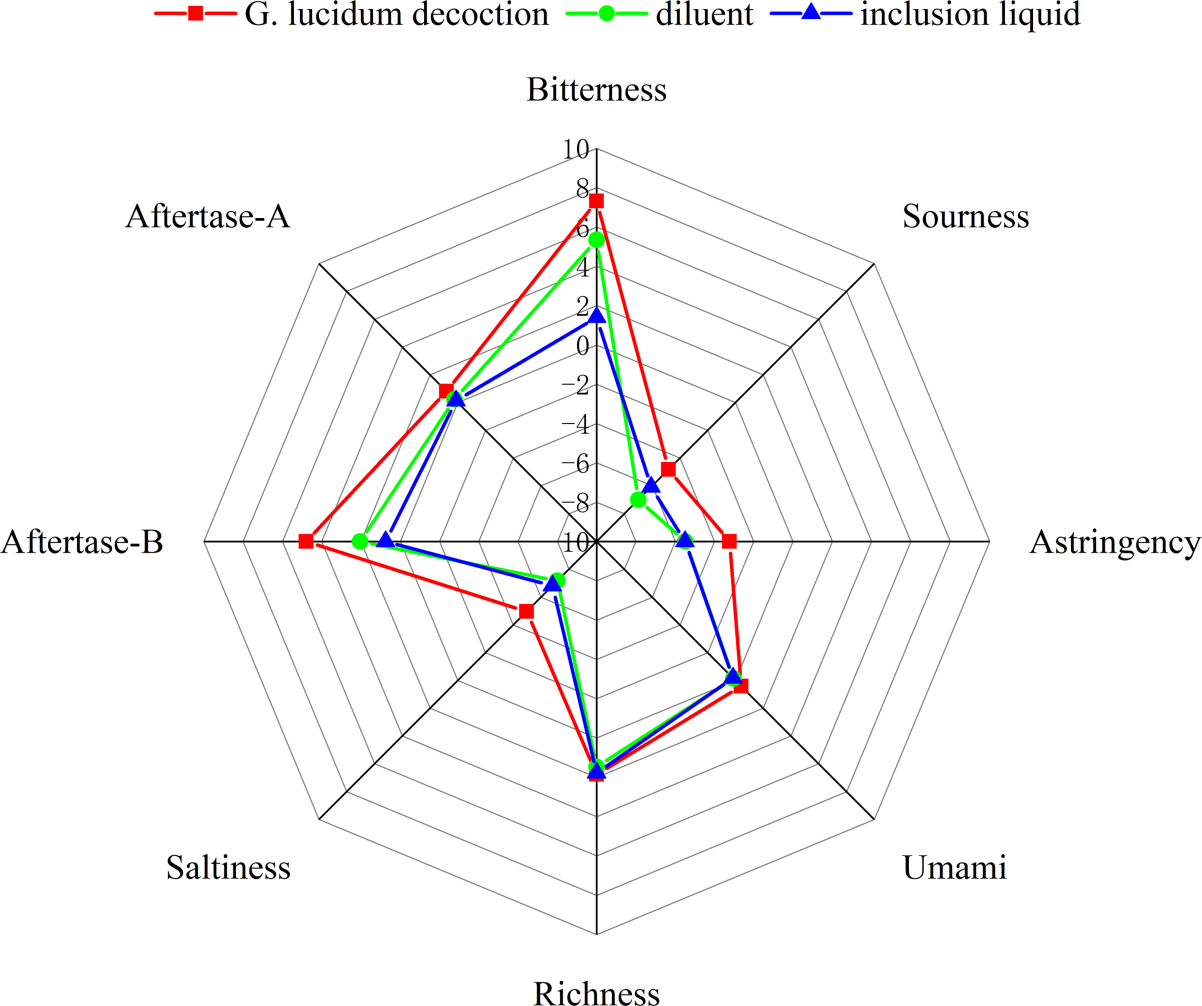
The results of the taste radar map.

An electronic tongue system was used to evaluate the sensory characteristics of *G. lucidum* decoction, inclusion liquid, and diluent. The taste attributes were evaluated before and after encapsulation with 2-HP-*β*-CD. The results presented in Fig. 2 demonstrate significant modifications in the bitterness, bitter taste, and aftertaste of the inclusion liquid after encapsulation with 2-HP-*β*-CD. No significant changes were observed in the individual taste components of the diluted solution. These findings suggest that the presence of 2-HP-*β*-CD primarily contributes to a substantial reduction in the bitter taste of *G. lucidum* decoction.

### Characterization analysis

#### Fourier Transform Infrared Spectroscopy analysis

**Figure 3:**
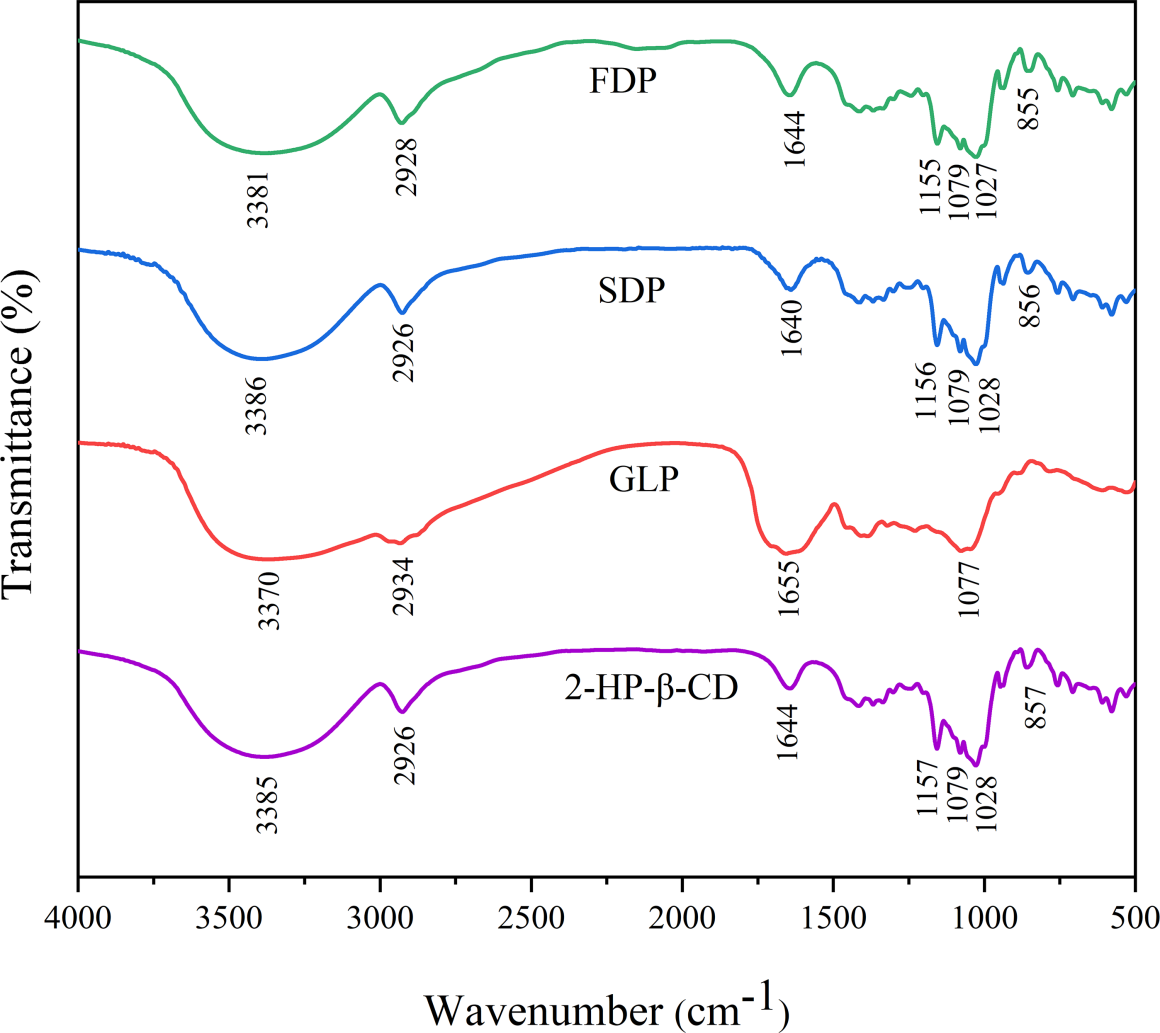
The results of FTIR spectra.

FTIR analysis is a commonly used technique in chemistry to investigate the chemical structure and interactions of compounds (Cui, Siva & Lin, 2019). The data obtained from FTIR can provide valuable information for establishing the formation of inclusion complexes based on the shift, appearance, or disappearance of characteristic peaks (Hadian et al., 2023). Figure 3 displays the FTIR spectra of 2-HP-*β*-CD, GLP, FDP and SDP in the wavelength range of 400–4,000 cm^−1^. The absorption peaks at 3,385 cm^−1^ and 2,926 cm^−1^ correspond to O-H and C-H bond stretching vibrations, respectively (Liu et al., 2022b; Venuti et al., 2019). The peak at 1,644 cm^−1^ indicates the stretching vibration of the C =O bond (Venuti et al., 2019). Additionally, symmetrical and asymmetrical stretching vibrations of *C* − *O* − *C* bonds were observed at 1,157 cm^−1^ and 1,028 cm^−1^, respectively (Liu et al., 2022a). Furthermore, the presence of an *α*-glycosidic linkage within the structure of 2-HP-*β*-CD was confirmed by a distinct band at 851 cm^−1^ (Sun et al., 2022). FTIR spectrum of HP-*β*-CD agreed well with the literature results (Imam et al., 2020; Liu et al., 2022a; Venuti et al., 2019). The ICs spectrum was similar to that of *β*-CD, and compared to that of *β*-CD, the intensity peaks in the spectrum of the inclusion complex were decreased and shifted, which may be evidence of the successful formation of the inclusion complex.

#### Proton nuclear magnetic resonance spectra analysis

Nuclear magnetic resonance (NMR) spectroscopy is commonly used to analyze chemical structures and investigate interactions between 2-HP-*β*-CD and guest molecules in ICs (Celebioglu & Uyar, 2020; Hadian et al., 2023). By observing changes in chemical shift (Δ*δ*) using ^1^D NMR, the incorporation of the bitter substance into the cavity of 2-HP-*β*-CD can be confirmed (Deshaware et al., 2018). This can be calculated as Δ*δ* = *δ*_(complex)_ -*δ*_(free)_. The results are presented in Table 5. The 2-HP-*β*-CD spectrum exhibited several prominent peaks ranging from 3.37 to 4.82 ppm. H1 (*δ* = 4.82 ppm), H2 (*δ* = 3.41 ppm), and H4 (*δ* = 3.37 ppm) were located on the outer region of the 2-HP-*β*-CD cavity, while H3 (*δ* = 3.74 ppm) and H5 (*δ* = 3.48 ppm) were inner protons within the 2-HP-*β*-CD cavity. These protons are crucial in investigating the interaction between guests and 2-HP-*β*-CD. H6 (*δ* = 3.52 ppm) is positioned along the edge of the cavity. The protons inside the 2-HP-*β*-CD (H3, H5 and H6) have greater mobility than those on its surface (H1, H2 and H4) when exposed to bitter compounds, as shown by their Δ*δ* values. The H3, H5, and H6 signals exhibited a downfield shift of 0.04, 0.03, and 0.02 for FDP and 0.05, 0.01, and 0.02 for SDP, respectively. The change observed was consistent with the literature (Deshaware et al., 2018; Hadian et al., 2023). The shift in proton signals in the cavity suggests the formation of a partial IC.

**Table 5:** The chemical shift (ppm) values of proton NMR spectra of 2-HP-*β*-CD and ICs.

H	2-HP-*β*-CD	FDP: 2-HP-*β*-CD		SDP: 2-HP-*β*-CD	
	*δ*_(2−HP−*β*−CD)_ (ppm)	*δ*_(FDP)_ (ppm)	Δ*δ*_1_ (ppm)	*δ*_(SDP)_ (ppm)	Δ*δ*_2_ (ppm)
H-1	4.82	4.83	0.01	4.83	0.01
H-2	3.41	–	–	3.41	0
H-3	3.74	3.70	−0.04	3.69	−0.05
H-4	3.37	3.34	−0.03	3.37	0
H-5	3.48	3.49	0.01	3.47	−0.01
H-6	3.52	3.50	−0.02	3.54	−0.02

**Notes.**

Δ*δ*_1_ = *δ*_(FDP)_ − *δ*_(2−HP−*β*−CD)_; Δ*δ*_2_ = *δ*_(SDP)_ − *δ*_(2−HP−*β*−CD)_.

### Physical characteristic analysis

#### Moisture content

The moisture level of the dried product is critical for its effectiveness, quality, range of applications, technological suitability, and stability during storage (Rattes & Oliveira, 2007). Klaypradit and Fredes’ research (Fredes et al., 2018; Klaypradit & Huang, 2008), suggests that dry food products with moisture contents between 3% and 10% exhibit excellent stability during storage. The results in Table 6 show that the moisture content of the microcapsules produced through SD and FD fell below the specified limits, indicating an extended shelf-life for these microcapsules. However, it is important to note that the moisture content was lower in the SDP (2.91 ± 0.12%) compared to the FDP (8.40 ± 0.75%). This finding is consistent with previous studies by Rezende, Nogueira & Narain (2018) and Rodríguez-Cortina, Rodríguez-Cortina & Hernández-Carrión (2022), which also reported lower moisture levels in samples subjected to SD compared to FD. Furthermore, the lower moisture levels in SD may be attributed to the impact of higher temperatures during the process.

**Table 6:** The effect of drying method on powder characteristics.

Physical characteristics	GLP	Encapsulation methods	
		FDP	SDP
Moisture content (%)	6.58 ± 0.24^b^	8.40 ± 0.75^a^	2.91 ± 0.12^c^
Bulk density (g/mL)	0.20 ± 0.004^b^	0.12 ± 0.001^c^	0.26 ± 0.004^a^
Solubility (%)	94.92 ± 0.84^a^	96.70 ± 0.73^a^	96.26 ± 1.30^a^
Hygroscopicity (g/100g)	46.67 ± 1.53^a^	8.33 ± 0.58^b^	7.33 ± 0.58^b^

**Notes.**

Means with different lowercase display significant differences (*p* < 0.05).

GLP *G. lucidum* microcapsule powder prepared by spray drying FDP inclusion compound prepared by freeze drying SDP inclusion compound prepared by spray drying

#### Bulk density

The characteristics of powders, including their packaging, transportation, storage, flow, and shelf-life stability, can be influenced by their bulk and tapped densities (Yaman et al., 2023). It is generally unfavorable to have low bulk density due to the increased package volume and air entrapment between particles, which can cause oxidation and compromise storage stability (Čulina et al., 2023; Goula & Adamopoulos, 2004). Table 6 shows that the bulk densities of SDP and FDP were 0.26 ± 0.004 g/mL and 0.12 ± 0.001 g/mL, respectively. There are significant differences in the existence of the two (*p* < 0.05). The study’s findings are consistent with those reported by Yaman et al. (2023). They demonstrated that the bulk densities of FDP (0.19 ± 0.001 g/mL) were significantly lower than those of SDP (0.29 ± 0.001 g/mL). Franceschinis et al. (2013) found that the pore size distribution of microcapsules is affected by the structural disparity between SD and FD, with FD preserving the crystal size and structure while SD may result in wall material degradation, leading to a more compacted product.

#### Solubility

The solubility of the powdered product in water is affected by several factors, with particular emphasis on the feed composition and particle size (Rezende, Nogueira & Narain, 2018). There was no significant difference in solubility between the MPs prepared by the two methods (*p* > 0.05) (Table 6). Due to the small particle size and the addition of highly soluble encapsulating agents, the sample presented a high average solubility, indicating a high capacity of the powder to remain homogeneous with water (Valente et al., 2019). This is because smaller particle sizes provide a larger surface area for hydration or facilitate better particle dispersion (Valente et al., 2019). The 2-HP-*β*-CD encapsulation system is a popular choice for improving solubility studies of substances that are difficult to dissolve due to its high solubility (Venuti et al., 2019). Above characteristics make the powder suitable for various applications, including beverages, dairy products, and instant consumables.

#### Hygroscopicity

According to research findings Rodríguez-Cortina, Rodríguez-Cortina & Hernández-Carrión (2022), MPs are considered highly moisture-absorbent when the hygroscopicity value exceeds 20%. Excessive levels of hygroscopy can cause powders to become sticky, compromising their long-term shelf stability. It is important to maintain appropriate levels of hygroscopicity to ensure product quality. In this study, we were able to decrease the hygroscopicity of the MPs that was prepared, resulting in a noteworthy enhancement of its shelf-life and storage stability. As shown in Table 6, the hygroscopicity range was measured to be between 7.33 g/100 g and 8.33 g/100 g. These results demonstrate that the MPs has superior hygroscopic properties compared to GLP (37.00 ±  1.00 g/100 g) (*p* < 0.05). This distinction can be attributed to several factors, including composition, type, carrier concentration, and microcapsule size. These factors collectively influence the overall level of moisture absorption (Tontul & Topuz, 2017).

### Antioxidant activity

Free radicals and reactive oxygen species are causative factors for various human pathologies. Antioxidant activity is significant due to its potential role in preventing or treating these conditions (Fan et al., 2020; Zeng et al., 2017). Because of the intricate reactivity of phytochemicals, a single method cannot accurately assess the antioxidant capacity of extracts (Zheng et al., 2020). To ensure reliability, it is advisable to use multiple test systems such as FRAP, DPPH, and hydroxyl radical analysis. Different approaches have unique mechanisms and limitations. Furthermore, the type of solvent employed also has an impact on the results of antioxidant activity (Ahmed et al., 2022). The extraction process with chosen solvents may contribute to overall antioxidant activities to varying degrees.

Table 7 summarizes the results, indicating significant differences in antioxidant properties among the experimental groups with different treatments (*p* < 0.05). The inclusion liquid resulted in a 20.27% increase in FRAP, a 30.01% increase in hydroxyl radical scavenging, and a 36.55% increase in DPPH scavenging compared to the diluted solution. These findings demonstrated that Improves antioxidant activity while debittering.

**Table 7:** The antioxidant activity of *G. lucidum* decoction under various treatments.

Antioxidant activity	Samples	
	Diluent	Inclusion liquid
FRAP (µmol FeSO_4_/mL)	0.074 ± 0.011^b^	0.089 ± 0.005^a^
Hydroxyl radical (%)	32.66 ± 2.69^b^	42.46 ± 1.25^a^
DPPH (%)	46.08 ± 1.15^b^	62.92 ± 1.84^a^

**Notes.**

Different letters indicate significant differences (*p* < 0.05).

**Figure 4:**
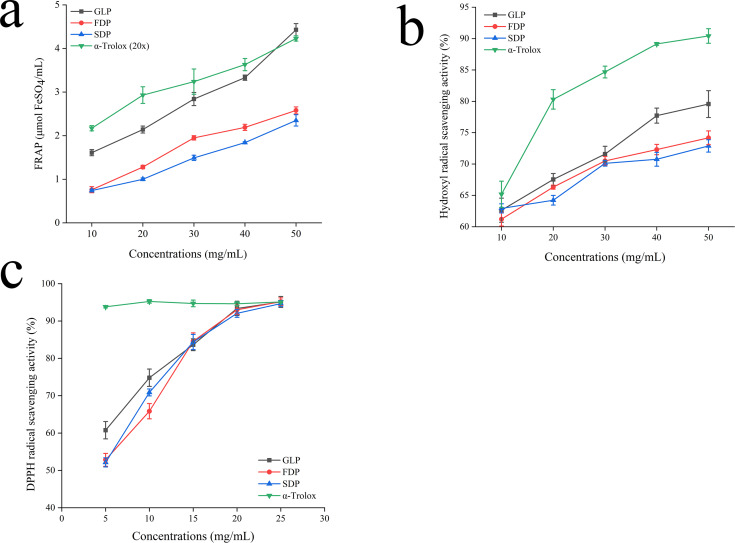
Antioxidant activity.

The FRAP assay is a commonly used method for assessing antioxidant activity by measuring the ability to reduce Fe^3+^ to Fe^2+^. Higher FRAP values indicate better antioxidant activity (Wang et al., 2019). The results showed a dose-dependent increase in both MPs and standards’ FRAP values (Fig. 4A). Notably, *α*-Trolox demonstrated significantly greater FRAP capacity compared to the MPs at high concentrations (10 mg/mL and 50 mg/mL). However, there was no significant difference in the FRAP values between FDP and SDP when compared at a concentration of 50.00 mg/mL (*p* > 0.05). At this concentration level, both MPs had significantly lower FRAP values than GLP and *α*-Trolox (*p* < 0.05). Prior, Wu & Schaich (2005) highlighted the limitations of FRAP in detecting substances that exert their effects through radical quenching mechanisms, such as thiols and proteins.

Previous studies have shown that the hydroxyl radical is highly reactive. Its scavenging activity can be inhibited by chelating ions like Fe^2+^ and Cu^2+^, which prevent its generation (Wang et al., 2010). The neutralization of the antioxidant function of the hydroxyl radical is crucial due to its high reactivity towards sugars, amino acids, lipids, and even nucleotides. Figure 4B shows that MPs, GLP, and *α*-Trolox can scavenge hydroxyl radicals. The efficacy of MPs in terms of antioxidant activity positively correlates with concentration. At a concentration of 50.00 mg/mL, trolox exhibited scavenging activity 1.14, 1.22, and 1.24 times higher than those observed for GLP, FDP and SDP, respectively.

The DPPH radical is commonly used to evaluate natural antioxidant properties because it can stabilize as a molecule when accepting an electron or hydrogen radical. This objective evaluation method is widely accepted in the field (Imam et al., 2020; Xie et al., 2020). Figure 4C illustrates that MPs exhibit dose-dependent scavenging activity against DPPH free radicals within a specific concentration range. No significant difference was observed in the scavenging capacity of MPs and GLP for DPPH. In the concentration range of 5–25 mg/mL, the scavenging capability of MPs was lower compared to that of *α*-Trolox. However, as the concentration increased, it approached that of the positive control and was not significantly different at 25 mg/mL.

## Conclusions

The objective of the present study was to optimize process conditions for the removal of bitterness using the RSM approach. The analysis results have shown that polynomial model with second order was sufficient for the prediction of response values with the change in inclusion conditions. The linear terms of the 2-HP- *β*-CD concentration and host-guest ratio, as well as the quadratic terms and interactive terms, were found to significantly affect the bitterness of the *G. lucidum* decoction. Analyses of the electronic tongue and antioxidant activity indicated that the formation of IC reduces bitterness and enhances the biological activity of *G. lucidum*, which can be attributed to the 2-HP-*β*-CD cavity effect. The results of FTIR and NMR spectroscopy demonstrated the successful formation of ICs. The physical properties of the MP were analyzed, and it was determined that spray drying was a more suitable method for this study. In conclusion, the results of the present study demonstrate that the ICs can effectively enhance food applications. Further research is needed to gain a full understanding of the potential risks and benefits associated with the incorporation of *G. lucidum* into the diet.

##  Supplemental Information

10.7717/peerj.17943/supp-1 Supplemental Information 1 Raw data

## Additional Information and Declarations

### Data Availability

The following information was supplied regarding data availability: The raw data is available in the Supplementary File.
